# Epidemiological drivers of turnip yellows virus and its vector *Myzus persicae* in Australian canola (*Brassica napus*) systems: a 3‐year study of commercial crops and noncrop vegetation

**DOI:** 10.1002/ps.71325

**Published:** 2026-09-20

**Authors:** Samantha E Edley, Owain R Edwards, Benjamin S Congdon, John G Black, Marielle Babineau, Anthony R van Rooyen, Alex Slavenko, Roger AC Jones, Andrew R Weeks, Paul A Umina

**Affiliations:** ^1^ Cesar Australia Brunswick VIC Australia; ^2^ Agriculture Victoria DEECA Mildura Office and Laboratory Irymple VIC Australia; ^3^ CSIRO Environment Floreat WA Australia; ^4^ Entozyme Pty Ltd Nedlands WA Australia; ^5^ Western Australian Department of Primary Industries and Regional Development Jandakot WA Australia; ^6^ In Vitro Technologies Noble Park North VIC Australia; ^7^ Department of Microbiology and Immunology, School of Veterinary Sciences The University of Melbourne Parkville VIC Australia; ^8^ UWA Institute of Agriculture, The University of Western Australia Perth WA Australia; ^9^ School of BioSciences, Bio21 Institute The University of Melbourne Parkville VIC Australia

**Keywords:** surveillance, virus disease, epidemiology, green peach aphid, integrated pest and disease management

## Abstract

**BACKGROUND:**

Turnip yellows virus (TuYV) is one of the most economically important viral pathogens of *Brassica napus* (canola) in Australia. However, the relative contributions of noncrop reservoirs, aphid vectors and virus genetic variation to TuYV epidemiology remain only partly resolved. *Myzus persicae* is the principal vector of TuYV and a major pest of *B. napus* production globally. In Australia, both *M. persicae* and TuYV persist between growing seasons in weeds and volunteer *B. napus* plants that can facilitate early‐season crop infection.

**RESULTS:**

We conducted a 3‐year study across southeastern and southwestern Australia to identify key noncrop reservoirs of *M. persicae* and TuYV, quantify seasonal and regional patterns of infection, characterize TuYV P5 variants, and assess predictors of TuYV occurrence in *B. napus* fields. *Arctotheca calendula* and *Raphanus raphanistrum* were identified as major reservoirs of both *M. persicae* and TuYV. P5‐II was the predominant virus genotype, accounting for >97% of TuYV‐positive samples. Disease symptoms were a reliable indicator of infection in *B. napus* during spring and earlier crop sowing dates were associated with increased TuYV incidence.

**CONCLUSION:**

The identification of key reservoir hosts and the strong association between earlier sowing and increased disease incidence highlight opportunities for targeted weed management, strategic sowing decisions and improved surveillance. These results provide new insights into the ecology of the *M. persicae*–TuYV–*B. napus* pathosystem, and support the development of integrated management strategies to reduce TuYV spread and minimize yield losses in Australian *B. napus* crops. © 2026 The Author(s). *Pest Management Science* published by John Wiley & Sons Ltd on behalf of Society of Chemical Industry.

## Graphical abstract

A 3‐year study across southern Australia aimed to characterize drivers of infection in the *Myzus persicae*–turnip yellows virus–*Brassica napus* pathosystem. Identification of key reservoir hosts and the strong association between earlier sowing and increased disease incidence highlight opportunities for targeted weed management, strategic sowing decisions and improved surveillance.

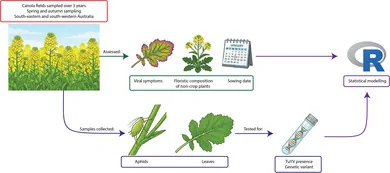

## 1 INTRODUCTION

The green peach aphid [*Myzus persicae* (Sulzer)] is one of the most destructive agricultural pests in the world owing to its ability to colonize plants from >40 families and transmit numerous economically important crop viruses.^1^, ^2^ Crop damage arises from direct feeding, feeding‐induced phytotoxicity, honeydew production that supports filamentous saprophytic ascomycetes, and most importantly from virus transmission.^2^, ^3^ Management historically has relied on synthetic chemical control,^2^ with phytosanitary, cultural and host resistance measures mainly applied at or before sowing.^4^, ^5^ However, insecticides are somewhat ineffective against nonpersistently transmitted viruses because they do not act quickly enough to prevent infection.^6^ Moreover, widespread and often indiscriminate insecticide use has driven *M. persicae* to become one of the most insecticide‐resistant pests worldwide.^5^, ^7^

In many countries, including Australia, turnip yellows virus (TuYV; species *Turnip yellows virus*, genus *Polerovirus*, family Solemoviridae; formerly beet western yellows virus) is the most widespread virus disease affecting oilseed brassicas such as canola (*Brassica napus*).^5^, ^8^ Infected *B. napus* plants typically exhibit chlorosis, yellowing or reddening of lower leaves, stunted growth and reduced biomass, although infections also may remain asymptomatic.^5^, ^8^, ^9^, ^10^, ^11^ High infection incidence at the rosette stage can cause yield losses of 47%, reduce oil content, and increase erucic acid and glucosinolate levels.^5^, ^8^ Losses are greatest when infection occurs in young seedlings, with postflowering infections rarely reducing yield or seed quality.^8^, ^12^, ^13^ Disease severity is influenced by plant growth stage at infection, temperature, virus strain, cultivar, and biotic or abiotic stress.^5^, ^10^, ^14^

TuYV is transmitted by aphids in a persistent, circulative and nonpropagative manner. The virus is acquired in minutes to hours, retained for the aphid's lifespan, and moves through the aphid's body to the salivary glands, but does not replicate within aphid cells.^6^, ^15^ After ~24 h of feeding, aphids typically become viruliferous and capable of transmission during phloem‐feeding.^6^, ^16^, ^17^ TuYV is restricted to the host plant phloem and primarily transmitted by *M. persicae*, which can achieve transmission efficiencies of >90%.^14^, ^18^ Alate aphids usually initiate primary TuYV spread by moving from infected to uninfected plant hosts,^19^ whereas both alate and apterous morphs contribute to secondary spread. Viruliferous apterae typically create localized infection foci, whereas alates can initiate secondary foci further away, facilitating wider dispersal across a crop field.^4^, ^5^, ^20^, ^21^

Within Australia, *M. persicae* and TuYV persist between *B. napus* growing seasons in infected weeds, crop volunteers and other brassica crops.^22^, ^23^ This ‘green bridge’ over the summer months is particularly common in southeastern (SE) Australia. The dry summer conditions in southwestern (SW) Australia restrict weed growth within crop fields, so both virus and aphid vectors mainly persist in wetter microclimates (e.g. drainage areas).^22^ Consequently, rather than a continuous ‘green bridge’, seasonal dynamics more closely resemble a ‘green ramp’, with annual weeds and volunteer *B. napus* emerging after autumn rainfall.^4^, ^5^, ^22^, ^24^, ^25^, ^26^

There are a large number of reservoir hosts of *M. persicae* and TuYV in Australia (i.e. noncrop plants that are a source of infection to new crops). Major hosts of *M. persicae* include *Raphanus raphanistrum* (wild radish), *Rapistrum rugosum* (turnip weed), *Myagrum perfoliatum* (muskweed), *Malva parviflora* (marshmallow), *Sonchus oleraceus* (common sowthistle) and *Arctotheca calendula* (capeweed).^5^, ^27^, ^28^, ^29^ Although host use varies among genotypes and clonal lineages, including the tobacco‐adapted *M. persicae nicotianae*,^30^, ^31^ recent evidence supports broad generalism rather than widespread plant‐family‐specific host races in *M. persicae*.^32^, ^33^ Surveys in SE Australia have detected TuYV in numerous weeds, including *R. raphanistrum*, *M. parviflora, S. oleraceus, A. calendula, Sonchus asper* (prickly sowthistle), *Senecio vulgaris* (groundsel), *Sinapsis arvensis* (wild mustard), *Capsella bursa‐pastoris* (Shepherd's purse) and *Erodium moschatum* (stork's‐bill).^34^ In SW Australia, TuYV has mostly been reported from *R. raphanistrum, A. calendula*, numerous vegetable brassicas and cool‐season legumes.^9^, ^35^, ^36^ This broad host range expands the potential size of the reservoir base, facilitating spread into autumn‐sown *B. napus* crops from a ‘green bridge’ or ‘green ramp’.^4^, ^22^, ^25^, ^34^, ^37^

Across Australia, summer and early‐autumn rainfall typically increases *M. persicae* populations and dispersal into young *B. napus* crops, leading to higher TuYV incidence and greater potential for yield loss, whereas limited pre‐sowing rainfall suppresses aphid numbers and reduces TuYV impact.^4^, ^25^, ^38^, ^39^ Even relatively small ‘off‐season’ reservoirs near cropping areas can initiate TuYV epidemics, with risk influenced by inoculum proximity and size, timing and abundance of *M. persicae* flights, rainfall, temperature, wind and crop‐sowing date.^4^, ^40^

TuYV populations in Australia exhibit substantial genomic diversity, particularly in open reading frame 5 encoding the readthrough domain of the minor coat protein (P5). Three P5 variants—P5‐I, P5‐II (a recombinant of I and III) and P5‐III—have been identified.^41^ Studies have shown that these variants differ in host range and transmission efficiency, with *M. persicae* capable of transmitting all three.^14^, ^42^ Given the diversity of noncrop hosts in Australia and the host range differences between variants detected among a relatively small number of host plant species, it is likely that additional TuYV variant‐specific hosts exist.^5^ Such variation may influence variant prevalence in *B. napus* crops, owing to differences in replicative fitness, transmissibility and virulence.^14^

In this study, we investigated the plant‐host dynamics of the *M. persicae*–TuYV–*B. napus* pathosystem across SW Australia and SE Australia over 3 years. Specifically, we aimed to: (i) identify key noncrop reservoirs of *M. persicae* and TuYV; (ii) quantify seasonal and geographic patterns of infection; (iii) characterize TuYV P5 variants and plant–aphid concordance; and (iv) examine predictors of TuYV occurrence in *B. napus* crops.

## 2 MATERIALS AND METHODS

### 2.1 Field sampling

Sampling was conducted during spring (early September to mid‐October) and autumn (mid‐April to late May) across 3 years (2021–2023) at multiple field sites in both SW Australia (Western Australia) and SE Australia [Victoria and southern New South Wales (NSW)]. Sites spanned multiple rainfall zones and comprised targeted and randomly selected locations in each region (Fig. 1). In SE Australia, initial targeted sites were locations where TuYV had been detected in *M. persicae* collected on sticky traps or *B. napus* plants in autumn 2021.^39^ In SW Australia, initial targeted sites were a combination of locations where TuYV had been detected in *M. persicae* collected in autumn 2021,^39^ and locations previously surveyed for TuYV and aphids in Western Australia.^4^ Random sites were selected based on their proximity to *B. napus* crops and surrounding vegetation, while ensuring broad spatial distribution across each region (~30 km apart in SE Australia and greater distances in SW Australia). In regions with compressed rainfall zones (e.g. Esperance in SW Australia), randomly selected sites were located closer together. Site selection differed in subsequent years—see Supporting Information Methods for details.

**Figure 1:**
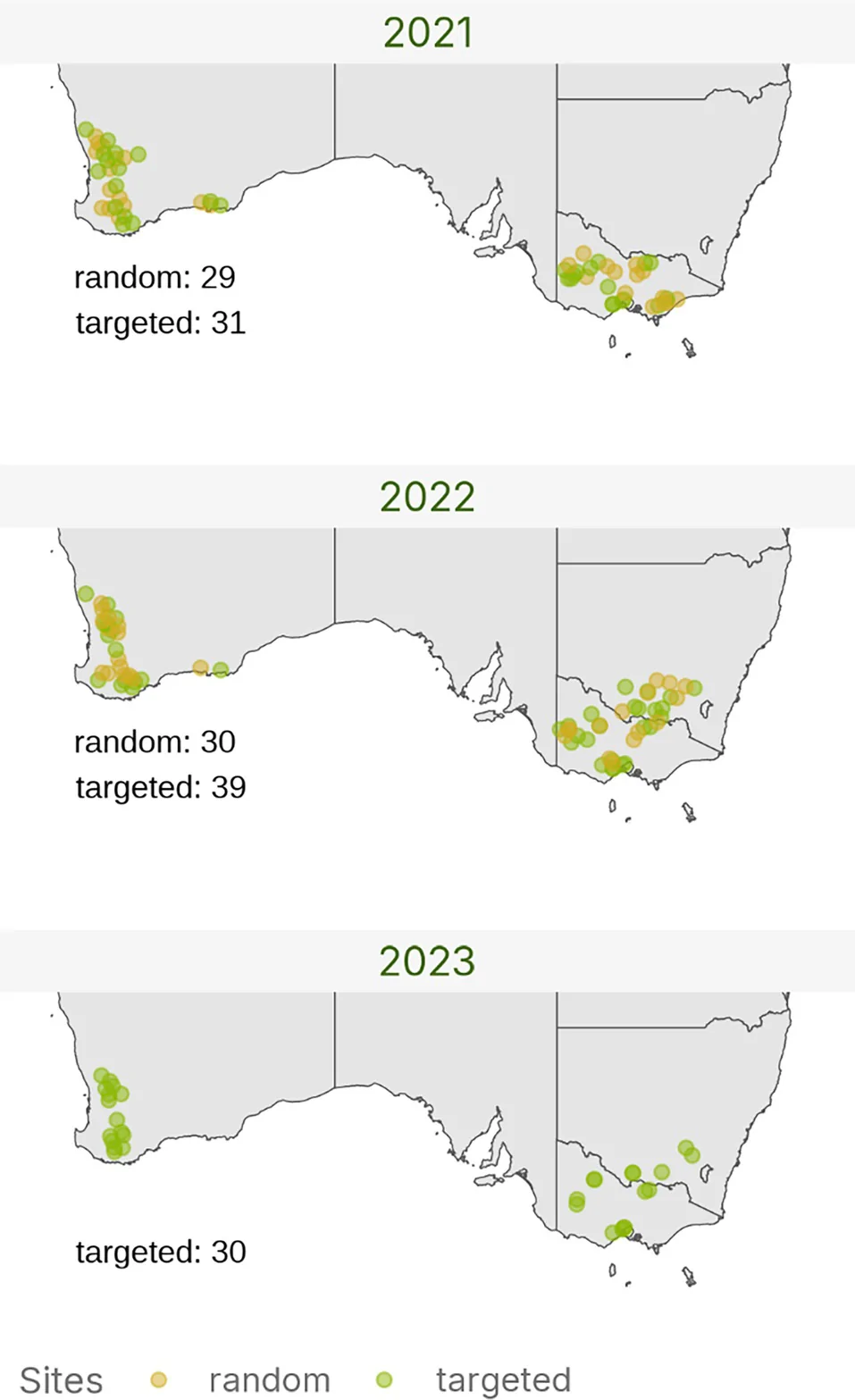
Geographic distribution of sampling sites across southern Australia from 2021 to 2023. The total number of random and targeted sites sampled each year is shown below each map.

At each site, transects were established along crop edges to sample for *M. persicae* and TuYV. Samples were taken from noncrop plants along each transect, with the number of sampled plants varying depending on the presence and abundance of species at each site. Floristic composition of noncrop vegetation (recorded to species or genus level), the presence or absence of visual disease symptoms on plant leaves, and the percentage ground coverage of each noncrop plant species were recorded. Plant coverage was subsequently calculated for each plant family as a proportion of the total noncrop vegetation (Fig. S1). Additionally, the foliage of sampled plants was thoroughly inspected for aphids. Aphid abundance was measured, and aphid and whole leaf samples were collected. At many of the sites, the adjacent *B. napus* crops also were sampled. Crop plants were sampled along transects that were directly adjacent to, and mirroring, the noncrop vegetation transects.

See Supporting Information Methods for full details of the sampling designs and sample storage protocols for each year.

### 2.2 Aphid identification and virus testing in aphid and leaf material

Aphids were morphologically identified to species level under a dissecting microscope.^1^ Adult aphids (both apterae and alates) were then separated into two categories: (i) *M. persicae* and (ii) other aphid species.

For the 2021 and 2022 collections, three adult *M. persicae* (either apterae or alates) were randomly selected from each plant and RNA was extracted individually using AllPrep DNA/RNA mini kits (Qiagen, Clayton, Australia) according to the manufacturer's protocol. For the 2023 collections, three adult *M. persicae* (either apterae or alates) were randomly selected from each plant and pooled before extraction using an RNeasy mini kit (Qiagen) according to the manufacturer's protocol, to reduce the screening time. RNA from each leaf sample was extracted using an RNeasy Plant mini kit (Qiagen) according to the manufacturer's protocol.

All extracts were tested for TuYV using a PrimeTime quantitative reverse‐transcription (qRT)‐PCR assay,^39^ using forward primer TuYV‐F (5′‐ GTTGCCGAGGACCAATTCA −3′), reverse primer TuYV‐R (5′‐ GGTGATTCTGAAAGAACCAGCTA −3′) and probe TuYV‐P (5′‐/56‐FAM/CCTCTACAA/ZEN/AGGCAATGGTTCTTCATCGA/3IABkFQ/‐3′). Assays were performed in triplicate using a LightCycler 480 II system (Roche, Basel, Switzerland) in a 384‐well format. Each 10‐μL reaction contained 2.5 μL Taqman Fast Virus 1‐step MasterMix (4×), 0.25 μL of PrimeTime assay (40×), 5.25 μL RNase‐free water and 2 μL RNA template. Cycling conditions consisted of a reverse transcriptase step of 50 °C for 5 min, RT inactivation / initial denaturation at 95 °C for 20 s, followed by 50 cycles of 95 °C for 3 s and 60 °C for 30 s.

Known TuYV positive (TuYV+) and TuYV negative (TuYV–) samples were included in each assay plate. Amplification profiles were analyzed using the Absolute Quantification module of the LightCycler 480 II software package to determine cycle quantification (Cq) values. The mean of the triplicate cycle threshold (Ct) value was used to determine TuYV presence. Samples with Ct values >35 were considered negative, and those with Ct values ≤35 were considered positive. TuYV+ control samples were sourced from the Western Australian Department of Primary Industries and Regional Development.

All samples identified as TuYV+ were subsequently tested using a multiplex PrimeTime qRT‐PCR designed to distinguish among the three P5 variants of TuYV (Table 1), using the same cycling conditions and Ct threshold described above.

**Table 1:** Primers and probes used in multiplex PrimeTime qRT‐PCR assays to differentiate the three TuYV P5 variants.

Assay name	Component	Sequence (5′–3′)
TuYV_Group_1_FAM	Forward primer	TCCTCCTTGACTTCCTCCTT
	Reverse primer	CCTTGCTGGCGCTTTATTTG
	Probe	/56‐FAM/AGCTCACTA/ZEN/CAAGTGAAAGGGCGA/3IABkFQ/
TuYV_Group_2_HEX	Forward primer	CAAGGACAGTGTCTGGAAAGAG
	Reverse primer	GACAGCGGGTCCAACAATAA
	Probe	/5HEX/AACAACTGG/ZEN/TTGATGACGCCTCCTT/3IABkFQ/
TuYV_Group_3_Cy5	Forward primer	GCCTCCGGTACATTGAGAAT
	Reverse primer	CGGGACTGGAAACACATACA
	Probe	/5Cy5/ACTTGAATA/TAO/CCATCGGGCGTCCAC/3IAbRQSp/

### 2.3 Statistical analyses

For most research questions, the response variable was binary (TuYV/*M. persicae* presence or absence) and analyses were therefore conducted using binomial logistic regression models (presence = 1 and absence = 0). To assess the contribution of random effects (sampling site and year of sampling), two intercept‐only null models were fitted in R v4.2.1^43^ for each dataset (see below): (i) an intercept‐only generalized linear model (GLM) with a binomial family and canonical logit link function using the ‘glm’ function, and (ii) a generalized linear mixed‐effects model (GLMM) including site and year as random effects (binomial family and canonical logit link function) using the ‘glmer’ function from the lme4 package v1.1.30.^44^ The ‘pchisq’ function was used to calculate the *P*‐value from a model likelihood ratio (MLR) test comparing the two models. Inclusion of random effects significantly improved model fit for most response variables (*P* < 0.001), and therefore all downstream models included site and year as random effects unless otherwise specified. Geographical region (SW Australia and SE Australia) and season (autumn and spring) were also included as covariates unless specified otherwise. Different research questions used different filtered subsets of the full dataset and sets of predictor variables considered. These are detailed in the Supporting Information Methods.

Exhaustive model selection was performed for each research question across marginal models containing all possible combinations of predictor variables and their two‐way interactions using the ‘glmulti’ function from the glmulti package v1.08.^45^ Models were ranked using the Bayesian Information Criterion (BIC), and the model with the lowest BIC was selected.^46^, ^47^ The fit of the best model relative to an intercept‐only model was assessed using a MLR test using the ‘anova’ function. Marginal effects and 95% confidence intervals (CIs) were visualized using the ‘plot_predictions’ function from the marginaleffects package v0.21.0.1.^48^ *Post hoc* comparisons were conducted using the ‘emmeans’ and ‘pairs’ functions from the emmeans package v1.8.2,^49^ with Benjamini & Hochberg correction applied to control for the false discovery rate.^50^

For GLMMs, effect sizes are reported as odds ratios (ORs) with 95% CIs. Marginal (*R* ^2^) values are reported to indicate the proportion of variance attributable to fixed effects.

## 3 RESULTS

### 3.1 Noncrop host plants

#### 3.1.1 *Noncrop hosts of* Myzus persicae

*Myzus persicae* was most frequently detected on Asteraceae (101 of 225, 44.9%), followed by Brassicaceae (96 of 225, 42.7%), with *A. calendula* (63 of 225, 28.0%) and *R. raphanistrum* (61 of 225, 27.1%) the most common noncrop host species (Fig. 2). In SE Australia, *M. persicae* was predominately found on *S. oleraceus* in autumn, and *A. calendula*, volunteer *B. napus* and *M. parviflora* in spring. In SW Australia, *M. persicae* was predominantly found on *A. calendula* and *R. raphanistrum* in spring (Fig. 2).

**Figure 2:**
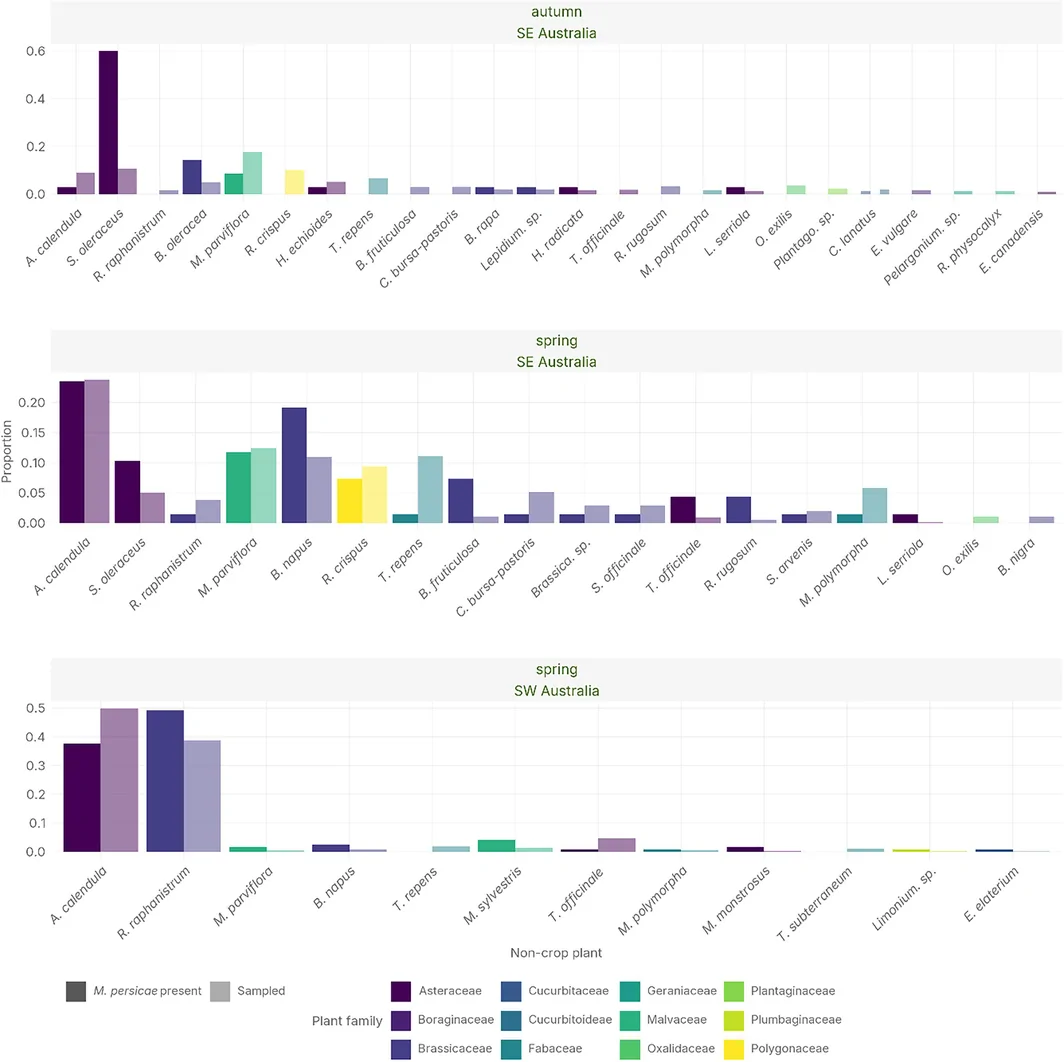
Occurrence of *M. persicae* on noncrop vegetation across sampling seasons (autumn and spring) and geographic regions (SE Australia and SW Australia). Dark bars represent the proportion of plants of each species (or genus) on which *M. persicae* was detected. Light bars represent the proportion of plants of each species (or genus) sampled. For visualization purposes, only plant taxa comprising ≥1% of samples and/or hosting *M. persicae* are shown. Note: *M. persicae* was not detected during autumn sampling in SW Australia.

Relative to sampling effort (i.e. the proportion of each plant type sampled), *M. persicae* incidence was significantly greater than expected on *S. oleraceus* during autumn in SE Australia (*χ* ^2^ = 105.36, df = 23, *P* < 0.001), on *R. raphanistrum* during spring in SW Australia (*χ* ^2^ = 44.50, df = 11, *P* < 0.001), and—despite limited sampling—on volunteer *B. napus* during spring in SE Australia, compared with other noncrop hosts (*χ* ^2^ = 84.44, df = 17, *P* < 0.001). In SE Australia, *M. persicae* occurrence was significantly higher on Asteraceae than on Fabaceae (OR = 27.22, 95% CI 3.05–243.00, *P* < 0.01), Malvaceae (OR = 3.90, 95% CI 1.28–11.93, *P* < 0.01) and Polygonaceae (OR = 6.22, 95% CI 1.42–27.28, *P* < 0.01), and higher on Brassicaceae than on Fabaceae (OR = 12.10, 95% CI 1.33–110.31, *P* < 0.05). In SW Australia, *M. persicae* occurrence was significantly lower on Asteraceae than on Brassicaceae (OR = 0.43, 95% CI 0.21–0.88, *P* < 0.05) and Malvaceae (OR = 0.08, 95% CI 0.02–0.47, *P* < 0.001). All other pairwise comparisons were nonsignificant (*R* ^2^ = 80.8%; Fig. S2).

#### 3.1.2 Noncrop hosts of TuYV

TuYV was most frequently detected in Asteraceae (391 of 970, 40.3%; mean Ct value = 31.6, range = 12.0–35.0) and Brassicaceae (1200 of 1753, 68.5%; mean Ct value = 26.7, range = 9.9–35.0), with *A. calendula* (360 of 692, 52.0%; mean Ct value = 31.4, range = 12.0–35.0) and *R. raphanistrum* (290 of 404, 71.8%; mean Ct value = 24.9, range = 9.9–34.9) among the most frequently infected noncrop species (Fig. 3). Other noncrop hosts commonly infected with TuYV included *M. parviflora* and *Oxalis exilis* (in SE Australia in autumn) and *T. repens* (in SW Australia in autumn).

**Figure 3:**
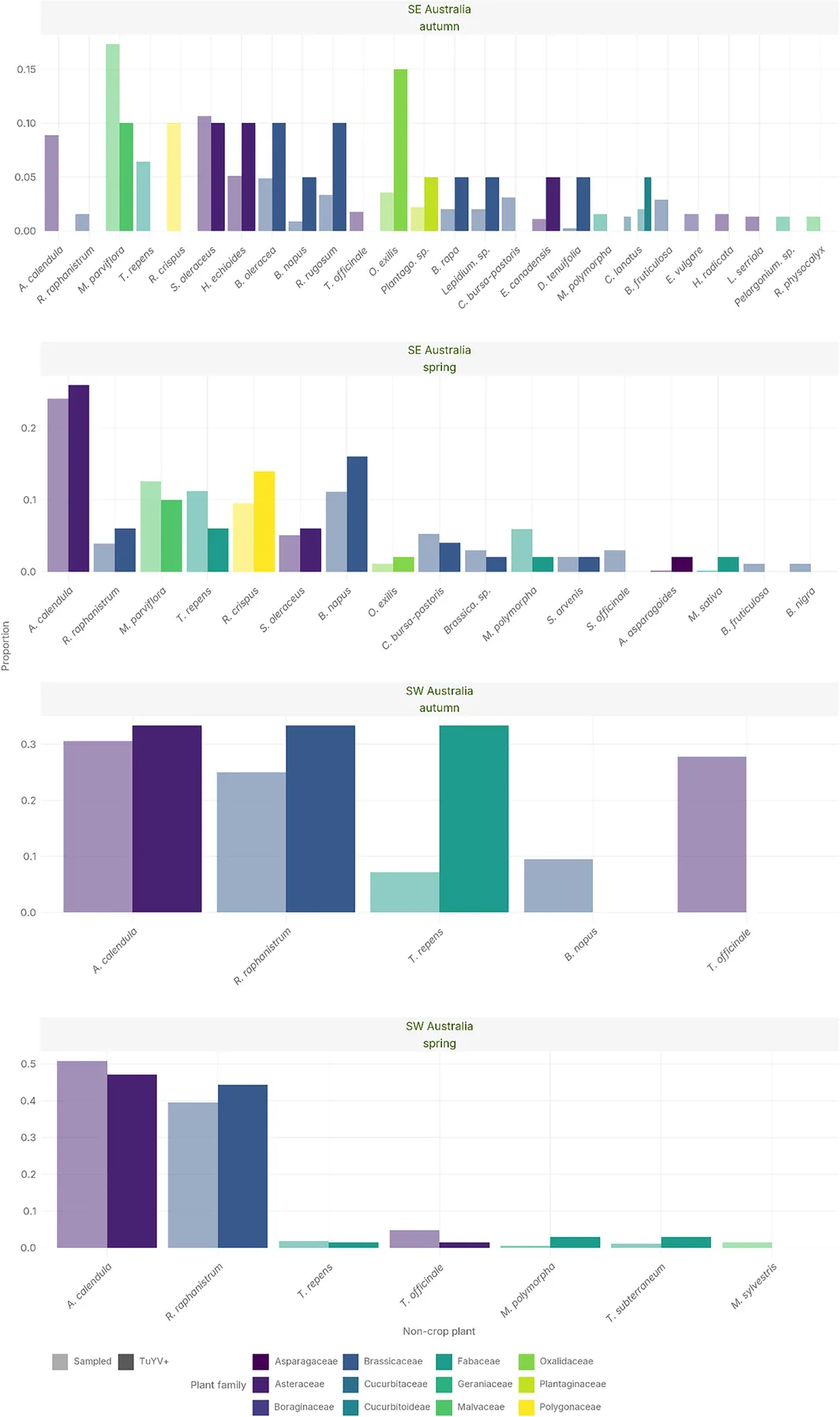
Occurrence of TuYV on noncrop vegetation across sampling seasons (autumn and spring) and geographic regions (SE Australia and SW Australia). Dark bars represent the proportion of plants of each species (or genus) on which TuYV was detected. Light bars represent the proportion of plants of each species (or genus) sampled. For visualization purposes, only plant taxa comprising ≥1% of samples and/or hosting TuYV are shown.

Relative to sampling effort, TuYV incidence was greater than expected on some noncrop host species, most notably *R. raphanistrum* across the study period (*χ* ^2^ = 112.01, df = 88, *P* < 0.05). Across both regions, TuYV occurrence was significantly higher on Brassicaceae than on Asteraceae (OR = 3.83, 95% CI 2.24–6.54), Fabaceae (OR = 2.84, 95% CI 1.36–5.92), Malvaceae (OR = 3.48, 95% CI 1.55–7.81) and Polygonaceae (OR = 6.21, 95% CI 2.09–18.51; *P* ≤ 0.001 in all cases), but not Oxalidaceae (OR = 3.84, 95% CI 0.41–36.03, *P* = 0.52). All other pairwise comparisons between plant families were nonsignificant (*R* ^2^ = 51.5%; Fig. S3).

A complete list of host plants surveyed in this study can be found in Table 2.

**Table 2:** Host plants and associated TuYV P5 variants detected in this study

Family	Species	Common name	TuYV P5 variant		
			P5‐I	P5‐II	P5‐III
Asparagaceae	*Asparagus asparagoides*	Bridal creeper	N (0/1)	Y (1/1)	N (0/1)
Asteraceae	*Arctotheca calendula*	Capeweed	Y (5/692)	Y (317/692)	Y (4/692)
	*Sonchus oleraceus*	Sowthistle	N (0/98)	Y (6/98)	N (0/98)
	*Taraxacum officinale*	Dandelion	N (0/104)	Y (21/104)	N (0/104)
Boraginaceae	*Echium vulgare*	Viper's bugloss	N (0/14)	Y (3/14)	N (0/14)
Brassicaceae	*Brassica fruticulosa*	Twiggy turnip	N (0/22)	Y (5/22)	N (0/22)
	*Brassica napus*	Canola (crop)	Y (120/1013)	Y (733/1013)	Y (27/1013)
	*Brassica napus*	Volunteer canola	Y (2/114)	Y (33/114)	Y (2/114)
	*Brassica nigra*	Black mustard	N (0/8)	Y (8/8)	N (0/8)
	*Brassica oleracea*	Cabbage	Y (1/25)	Y (1/25)	N (0/25)
	*Brassica rapa*	Field mustard	N (0/9)	Y (9/9)	N (0/9)
	*Brassica* sp.	Mustard	N (0/24)	Y (9/24)	N (0/24)
	*Capsella bursa‐pastoris*	Shepherd's purse	Y (3/56)	Y (12/56)	Y (2/56)
	*Lepidium* sp.	Peppergrass	N (0/19)	Y (4/19)	N (0/19)
	*Raphanus raphanistrum*	Wild radish	Y (8/405)	Y (274/405)	Y (3/405)
	*Rapistrum rugosum*	Turnipweed	N (0/19)	Y (7/19)	N (0/19)
	*Sinapsis arvensis*	Mustard	N (0/15)	Y (3/15)	N (0/15)
	*Sisymbrium officinale*	Hedge mustard	N (0/22)	Y (3/22)	Y 2(/22)
Cucurbitaceae	*Ecballium elaterium*	Squirting cucumber	N (0/1)	Y (1/1)	N (0/1)
Fabaceae	*Medicago polymorpha*	Burr medick	Y (1/58)	Y (9/58)	N (0/58)
	*Medicago sativa*	Alfalfa (Lucerne)	N (0/4)	Y (1/4)	N (0/4)
	*Trifolium repens*	White clover	Y (2/141)	Y (25/141)	N (0/141)
	*Trifolium subterraneum*	Subterranean clover	N (0/14)	Y (9/14)	N (0/14)
Malvaceae	*Malva parviflora*	Mallow	Y (2/195)	Y (33/195)	Y (6/195)
Oxalidaceae	*Oxalis exilis*	Wood sorrel	Y (1/27)	Y (1/27)	N (0/27)
Polygonaceae	*Rumex crispus*	Dock	N (0/119)	Y (10/119)	N (0/119)

Only species testing positive for at least one variant are shown. Variant presence is indicated as positive (‘Y’) or negative (‘N’). Values in parentheses indicate the number of positive samples relative to the total number tested.

### 3.2 Temporal and spatial patterns

#### 3.2.1 Seasonal differences

*Myzus persicae* occurrence on noncrop hosts did not differ significantly between seasons (autumn and spring) in SE Australia (OR = 0.85, 95% CI 0.51–1.44, *P* = 0.55; Fig. S2). No aphids were detected in SW Australia in autumn surveys, precluding a statistical comparison with spring surveys where *M. persicae* were commonly detected.

Across all noncrop plant families, the probability of TuYV detection was significantly higher in spring than autumn in both regions (OR = 290.92, 95% CI 133.80–632.50, *P* < 0.001; Fig. S3).

#### 3.2.2 Regional differences

The probability of detecting *M. persicae* on noncrop plants during spring was significantly higher in SW Australia than in SE Australia for Malvaceae (OR = 23.03, 95% CI 4.92–107.84, *P* < 0.001) and Brassicaceae (OR = 2.57, 95% CI 1.15–5.73, *P* < 0.05). No *M. persicae* were detected across all SW Australian sites in autumn 2023, precluding assessment of regional differences for this season (Fig. S2). In spring, the probability of detecting TuYV in noncrop plants was significantly higher in SW Australia than in SE Australia (OR = 13.00, 95% CI 6.35–26.60, *P* < 0.001), whereas the opposite pattern was observed in autumn (OR = 0.12, 95% CI 0.03–0.53, *P* < 0.01; Fig. S3).

#### 3.2.3 Crop *versus* noncrop plants, and floristic composition

Across both regions, TuYV was detected more frequently in *B. napus* crops than in noncrop plants in spring (OR = 4.97, 95% CI 3.80–6.49, *P* < 0.001; *R* ^2^ = 22.7%). Neither the probability of detecting *M. persicae* nor the probability of detecting TuYV in *B. napus* crops differed with the degree of coverage of different noncrop plant families along crop borders. In both cases, the null model was the best supported, while models including plant coverage as a fixed effect had BIC weights of <1% and <5% for *M. persicae* detection and TuYV detection, respectively.

Variant P5‐I was detected more frequently in *B. napus* crops than in noncrop plants (OR = 5.45, 95% CI 3.39–8.75, *P* < 0.001), whereas noncrop plants were more likely to be infected with P5‐II than *B. napus* crops (OR = 2.42, 95% CI 1.20–4.91, *p* < 0.05; *R* ^2^ = 81.3%).

#### 3.2.4 B. napus sowing date

Earlier sowing dates were associated with a higher probability of detecting TuYV in *B. napus* crops in both regions (OR = 0.93 per day, 95% CI 0.88–0.98, *P* < 0.01; *R* ^2^ = 16.8%), with overall TuYV incidence higher in *B. napus* crops in SW Australia than in SE Australia at all sowing dates (OR = 18.54, 95% CI 3.63–94.63, *P* < 0.001) (Fig. 4).

**Figure 4:**
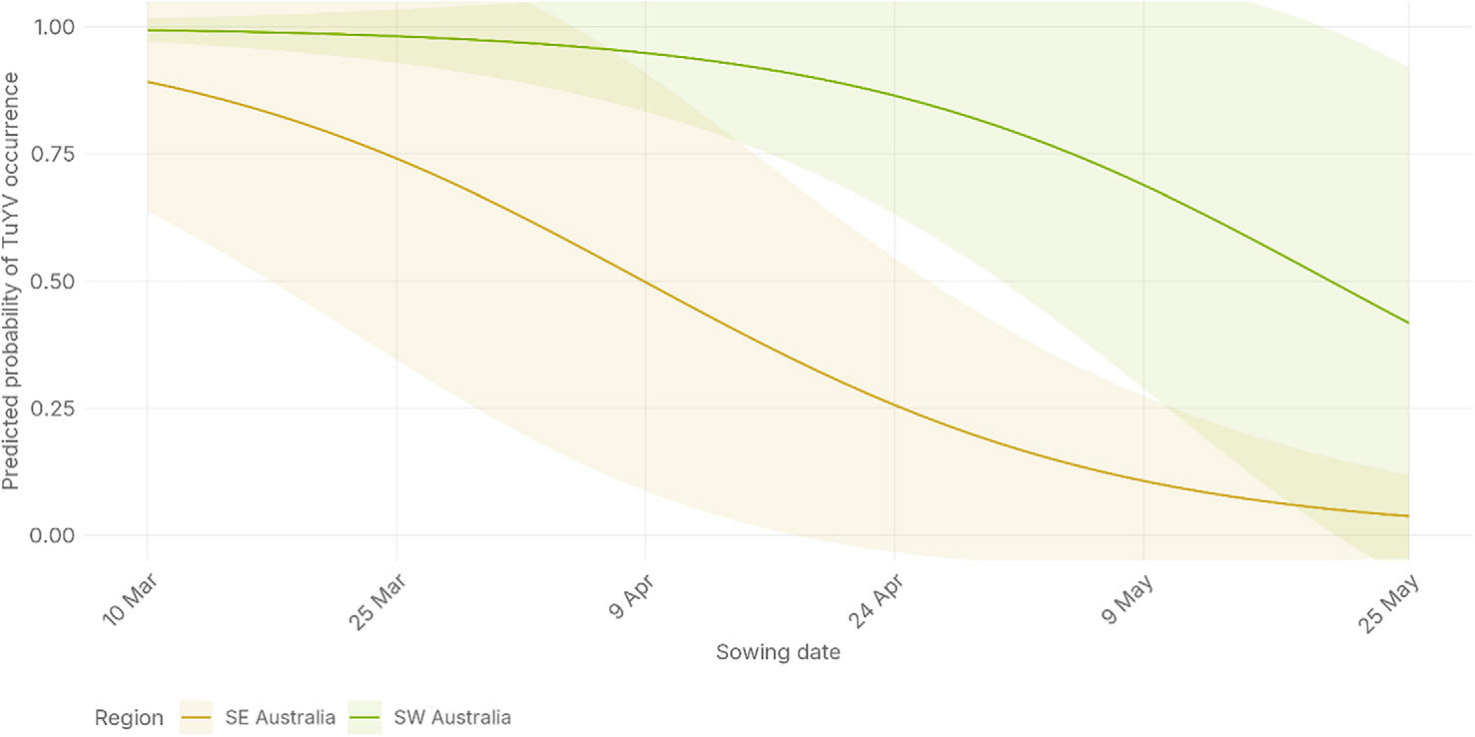
Predicted probability of TuYV occurrence in *B. napus* crops in relation to sowing date across geographic regions (SE Australia and SW Australia).

### 3.3 TuYV P5 variants

#### 3.3.1 TuYV P5 variant prevalence and coinfection

All three TuYV P5 variants were detected in both regions across all years, except for P5‐III, which was not detected in SW Australia in 2023. Across all TuYV+ samples (aphid and leaf samples), P5‐III was more prevalent in SE Australia than in SW Australia (Fig. 5).

**Figure 5:**
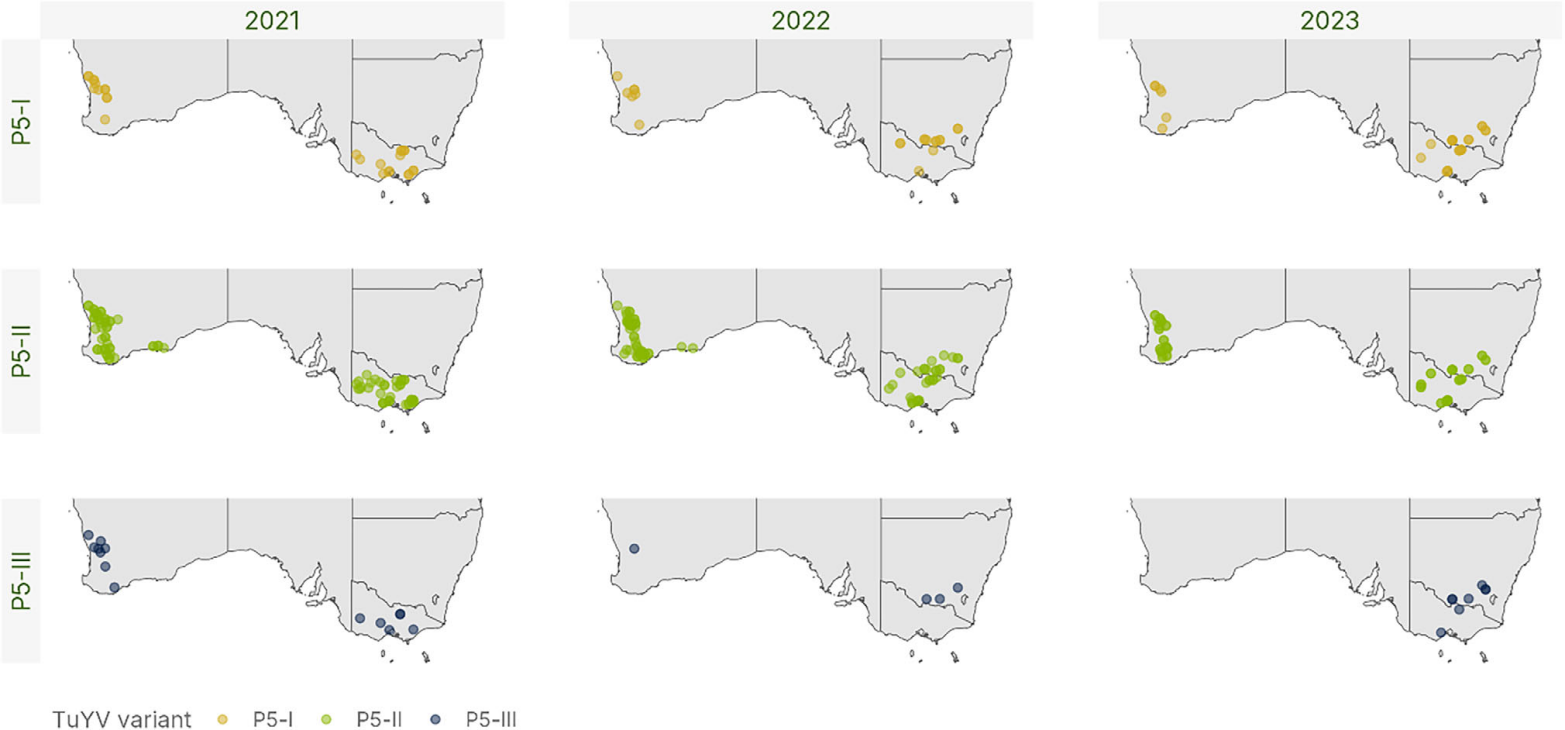
Geographic distribution of TuYV P5 variants detected in *M. persicae,* noncrop and crop (*B. napus*) plants in southern Australia from 2021 to 2023. Data from autumn and spring sampling are combined.

P5‐II was the most prevalent variant overall, accounting for 97.1% of all TuYV+ plants and 96.9% of all TuYV+ aphids for which variant identity could be determined (Table 2). Only 9.2% and 2.9% of all TuYV+ plants were variant P5‐I and P5‐III, respectively. Only 18.2% and 0.4% of all TuYV+ *M. persicae* were variant P5‐I and P5‐III, respectively. Across both *B. napus* crops and noncrop plants, P5‐II was more prevalent than P5‐I (OR = 1172.25, 95% CI 592.88–2317.80) and P5‐III (OR = 3312.38, 95% CI 1570.44–6986.46) and P5‐I was more prevalent that P5‐III (OR = 2.83, 95% CI 1.73–4.61; *P* < 0.001 in all cases; *R* ^2^ = 81.3%).

Coinfection of P5 variants was common in aphid and leaf samples. 80.4% of all plants infected with variant P5‐III also were infected with P5‐II. Likewise, 73.1% of all plants infected with variant P5‐I were coinfected with P5‐II. Eleven plants (0.7%; three noncrop and eight crop plants) carried all three variants, all collected during spring across the three study years. Interestingly, all autumn noncrop plants detected with TuYV (*n* = 29) were infected with only a single P5 variant (26 P5‐II, three P5‐I and no P5‐III detections).

Among *M. persicae* infected with variant P5‐I, 84.0% also were infected with P5‐II. Variant P5‐III was extremely rare in *M. persicae* across the study period, occurring in only two aphids, both of which were also coinfected with P5‐II. One of these aphids was also infected with P5‐I, representing the only individual to carry all three variants. Similar to autumn plant samples, *M. persicae* collected in autumn were almost exclusively infected with variant P5‐II only, except for one individual which was infected with both P5‐I and P5‐II.

#### 3.3.2 Plant–aphid TuYV P5 variant concordance

Overall, there was strong concordance between the TuYV P5 variant detected within *M. persicae* and the P5 variant detected in the host plant; 77.4% concordance for P5‐II and 50.0% for P5‐I (*χ* ^2^ = 73.40, df = 4, *P* < 0.001). However, variant mismatches between aphids and their host plant were relatively common on both noncrop and *B. napus* crop plants (Fig. S4). 44.2% of *M. persicae* infected with P5‐I were collected from plants infected only with P5‐II, and 18.2% of *M. persicae* infected with P5‐II were collected from plants infected only with P5‐I.

#### 3.3.3 TuYV P5 variant association with visual symptoms

The incidence of visual disease symptoms in *B. napus* crop plants was significantly higher in plants infected with variant P5‐I (48 of 120, 40.0%) than those infected with P5‐II (71 of 731, 9.7%) (*χ* ^2^ = 76.11, df = 1, *P* < 0.001; Fig. S5). The incidence of visual symptoms in *B. napus* crop plants infected with variant P5‐III was 18.5% (five of 27), which was not significantly different to either P5‐I (*χ* ^2^ = 3.53, df = 1, *P* = 0.06) or P5‐II (*χ* ^2^ = 1.37, *df* = 1, *P* = 0.24). The incidence of visual symptoms increased with the number of TuYV variants per plant (*χ* ^2^ = 78.41, df = 2, *P* < 0.001).

In noncrop plant hosts, there was no significant association observed between P5 variant and the incidence of visual disease symptoms (*χ* ^2^ = 0.69, df = 2, *P* = 0.71).

### 3.4 Predictors of TuYV

#### 3.4.1 TuYV detection in host plants and associated aphids

In spring, *M. persicae* was significantly more likely to be infected with TuYV when collected from TuYV+ host plants (noncrop and *B. napus* crop plants combined; OR = 3.23, 95% CI 1.67–6.25, *P* < 0.001; *R* ^2^ = 45.8%). This relationship could not be assessed in autumn as only three *M. persicae* specimens were collected from TuYV+ plants and all were infected with TuYV.

Across all noncrop and *B. napus* crop plants from which TuYV+ *M. persicae* were collected, 78.9% of plants were TuYV+ (37.5% in autumn and 81.0% in spring). Conversely, only 35.0% of plants from which TuYV– *M. persicae* were collected were infected with TuYV (0.0% in autumn and 51.7% in spring).

#### 3.4.2 Autumn *M. persicae* occurrence and TuYV incidence in spring

The presence of *M. persicae* in noncrop hosts during autumn did not predict TuYV incidence in adjacent *B. napus* crops in spring (null model preferred; likelihood ratio test *P* = 0.71).

#### 3.4.3 Visual symptoms as an indicator of TuYV infection

The presence of visual disease symptoms on plant leaves was strongly associated with the detection of TuYV in *B. napus* crop plants (OR = 10.73, 95% CI 3.63–31.72, *P* < 0.001; *R* ^2^ = 38.9%). Across the entire study period, > 85% of symptomatic *B. napus* crop plants were confirmed as TuYV+, whereas ~35% of asymptomatic plants also tested positive for TuYV. There was no significant association between visual symptoms and TuYV detection in noncrop host plants (OR = 1.42, 95% CI 0.77–2.61, *P* = 0.27).

## 4 DISCUSSION

Similar to many other countries, noncrop plant hosts in Australia serve as important reservoirs of both TuYV and *M. persicae*, facilitating primary virus spread into *B. napus* crops.^5^ Across 3 years and two regions, *M. persicae* was detected most frequently on Asteraceae and Brassicaceae, with *A. calendula* and *R. raphanistrum* consistently common hosts. These findings align with studies from India and elsewhere, where *M. persicae* commonly colonizes Asteraceae, Brassicaceae and Solanaceae hosts.^51^, ^52^ In autumn, *S. oleraceus* was the predominant host of *M. persicae* in SE Australia. Although noted as a host of *M. persicae*,^1^ it is rarely mentioned in the literature and was not identified as a host in a comprehensive study undertaken in parts of SW Australia.^22^ Furthermore, extracts of *S. oleraceus* have been reported to cause *M. persicae* mortality.^53^ The reasons underlying its apparent importance as an autumn host in SE Australia therefore remain unclear. In spring, *M. persicae* detections were highest on *A. calendula* in SE Australia and on *R. raphanistrum* in SW Australia. The prominence of *A. calendula* as a spring host has been reported previously in Australia, albeit in a different region,^27^, ^28^ while *R. raphanistrum* is recognized as a highly suitable host due to the high fecundity of *M. persicae* on this species, particularly for apterous morphs.^54^, ^55^

TuYV detections in noncrop vegetation were concentrated in Brassicaceae, with *A. calendula* and *R. raphanistrum* again among the most frequently infected species. These hosts therefore function dually as aphid host plants and virus inoculum reservoirs, supporting a ‘green bridge’ between growing seasons in SE Australia and a post‐summer ‘green ramp’ in SW Australia. The proximity of these species to emerging *B. napus* crops thus increases opportunities for early‐season virus transmission. However, it is important to note that TuYV epidemics are shaped by multiple interacting factors, including late summer and autumn rainfall, temperature, wind, crop sowing date, aphid abundance, crop plant density and length of the growing season.^4^, ^5^, ^25^, ^39^

Both *M. persicae* and TuYV were detected more frequently in spring than in autumn, consistent with previous Australian studies reporting greater aphid activity and TuYV incidence later in the growing season.^23^, ^56^ This pattern is likely to reflect the substantial population expansion that occurs over several months within crops and surrounding vegetation. In autumn, by contrast, most *B. napus* crops are only emerging or yet to be sown, and aphid and virus populations therefore depend more heavily on noncrop reservoirs. Nevertheless, when environmental conditions favour aphid survival and dispersal before (or soon after) sowing, widespread infection of young *B. napus* crops can occur, resulting in severe yield losses.^25^

Clear regional differences were also observed. In spring, *M. persicae* and TuYV were detected more frequently in SW Australia than in SE Australia, whereas aphid occurrence in autumn was greater in SE Australia. These differences are likely to be driven by contrasting climatic conditions. The grainbelt of SE Australia includes cooler temperate regions with more reliable year‐round rainfall, whereas SW Australia is dominated by Mediterranean climates characterized by hot, dry summers. Accordingly, aphids can be present year‐round in milder climates where summer rainfall maintains a continuous ‘green bridge’ of host plants. In hotter regions affected by summer drought, aphids are restricted to localized wet refuges such as roadside ditches and drainage areas supporting weeds and volunteer crops.^22^ Following autumn rainfall, these refuges may facilitate the development of a broader ‘green ramp’, enabling rapid aphid population growth and dispersal into newly emerging crops.^4^, ^5^ The very low abundance of *M. persicae* in SW Australia during autumn 2023 is likely to have reflected the exceptionally low rainfall recorded in that region during the summer–early autumn period. Previous work has shown that rainfall and wind exert stronger influences on *M. persicae* movement in SW Australia than SE Australia, whereas temperature and daylength appear to be more important drivers in SE Australia.^39^

Regional variation also was evident in the importance of individual weed hosts. *Raphanus raphanistrum* was confirmed as a key host in both regions, whereas *R. rugosum* was found to be an important host only in SE Australia, consistent with earlier studies (reviewed by Jones *et al*.^5^). By contrast, *M. parviflora*, previously considered important mainly in subtropical eastern Australia,^57^ was identified as a host in both regions in the present study. Together, these findings highlight the strong influence of regional climate and weed community composition on the epidemiology of the *M. persicae*–TuYV–*B. napus* pathosystem in Australia.

*Brassica napus* crops consistently exhibited higher TuYV prevalence than adjacent noncrop vegetation, reflecting both their high susceptibility to infection and their attractiveness to *M. persicae*. Once introduced into crops, the monoculture of highly suitable brassicaceous hosts is likely to facilitate rapid aphid feeding and virus transmission. Most *B. napus* cultivars are highly susceptible to TuYV and readily colonized by *M. persicae*,^13^ which is likely to explain the greater virus incidence observed in crops than in surrounding vegetation. Although crop cultivar information was recorded at each site wherever possible, cultivar‐specific analyses were not undertaken owing to the large number of cultivars represented and relatively small sample sizes in our study. The proportional coverage of noncrop plant families along crop borders did not predict *M. persicae* or TuYV occurrence within adjacent crops. This suggests that the presence and proximity of key reservoir hosts may be more important than their overall abundance, reinforcing the value of targeted pre‐sowing weed management, particularly of species such as *A. calendula* and *R. raphanistrum*. Previous studies have shown that weed control before sowing can substantially reduce TuYV disease pressure in *B. napus* crops.^5^, ^51^

TuYV incidence declined with later sowing dates, with infection risk consistently higher in SW Australia than SE Australia. Earlier sowing extends the period during which young seedlings are exposed to viruliferous aphid flights, thereby increasing the likelihood of primary infection.^23^, ^58^ In SE Australia in particular, *M. persicae* flights are less likely later in the season when temperatures decrease,^39^ highlighting one benefit of delayed sowing. However, delayed sowing may expose seedlings to unfavourable growing conditions or increase vulnerability to other pests, such as the redlegged earth mite (*Halotydeus destructor*).^59^, ^60^ Given the trend toward earlier sowing of *B. napus* crops across Australia, growers should account for the associated increase in TuYV risk.

*Myzus persicae* collected from TuYV+ plants were significantly more likely to be viruliferous than aphids collected from TuYV‐ plants. Nevertheless, occasional mismatches occurred, with TuYV+ aphids detected on TuYV‐ plants and *vice versa*. These discrepancies may reflect delays between virus acquisition or infection and detectable virus titres within plants or aphids, or reflect the recent arrival of migrating aphids from other hosts. High TuYV incidence in *A. calendula* and *R. raphanistrum* corresponded closely with high *M. persicae* occurrence, suggesting that these species may represent important reservoirs for subsequent TuYV infection of neighbouring *B. napus* crops. This potential transmission pathway could be tested experimentally by determining whether *M. persicae* can acquire TuYV from these hosts, subsequently feed on and transmit the virus to *B. napus*, and successfully colonize the crop, thereby facilitating further virus spread. More broadly, these findings highlight the mobility of aphid vectors and the temporal complexity of virus detection, supporting the value of testing both aphids and plants in surveillance programmes.

Detection of *M. persicae* in noncrop vegetation during autumn did not predict TuYV incidence in adjacent *B. napus* crops in spring. However, sampling was limited to a single crop border per site and did not account for other potential reservoirs within the surrounding landscape. Aphids may therefore have acquired virus from unsampled hosts before dispersal into spring crops. Although *M. persicae* alates are capable of long‐distance migration, their effectiveness as vectors declines with increasing dispersal distance compared with aphids originating from hosts near the crop.^61^ These findings suggest that interseasonal redistribution of aphids and the presence of unsampled reservoirs reduce the predictive value of localized autumn monitoring. Furthermore, even small numbers of incoming alates may carry TuYV,^39^ making aphid abundance alone an unreliable predictor of epidemic risk.

Visual disease symptoms were strongly associated with TuYV infection in *B. napus* crops during spring but not in noncrop hosts, many of which remained asymptomatic despite infection. This is consistent with previous studies demonstrating that TuYV symptoms are unreliable indicators of infection status, as symptoms may be confused with abiotic stress responses such as nutrient deficiency or herbicide damage, while infected plants may remain symptomless.^5^, ^62^ For example, *C. bursa‐pastoris* commonly exhibits yellowing and leaf curling following TuYV infection,^16^, ^63^ whereas infected *Vicia faba* (faba bean), *Pisum sativum* (field pea) and *Lens culinaris* (lentil) may show few or no visible symptoms despite measurable yield losses.^11^, ^35^ Likewise, TuYV‐associated yield losses in *B. napus* can occur without obvious foliar symptoms.^14^, ^17^ Consequently, molecular diagnostics should complement visual assessments, particularly in noncrop reservoirs where asymptomatic infection appears common.

This study also provides new insights into the distribution and prevalence of the three TuYV P5 variants across hosts, regions, and seasons. Variant P5‐II overwhelmingly predominated in both infected plants and viruliferous aphids, whereas P5‐I and P5‐III occurred at much lower frequencies. Similar dominance of P5‐II has recently been reported in South African *B. napus* crops.^64^ However, Congdon *et al*.^14^ found that a P5I isolate was transmitted more efficiently by *M. persicae* than P5‐II, suggesting a more complex relationship between TuYV genotype and fitness than can be explained by the P5 protein alone. All three P5 variants were detected in *B. napus* and *C. bursa‐pastoris*, consistent with Filardo *et al*.^41^ However, unlike previous studies, all three variants also were detected in *M. parviflora* and *R. raphanistrum*, whereas fewer variants were identified in *M. polymorpha* and *S. arvensis*. These findings suggest that the host ranges of the P5 variants are broader than previously recognized. Coinfection was common, particularly in *B. napus*, and a small number of plants harboured all three variants simultaneously.

In *B. napus* crop plants, disease symptoms were more frequent in infections involving P5‐I than P5‐II, and symptom incidence increased with the number of variants detected, suggesting a possible additive effect of coinfection. Glasshouse studies likewise reported more severe symptoms in *B. napus* plants infected with a P5‐I isolate than with P5‐II or P5‐III.^14^ However, interpretation is complicated by the frequent co‐occurrence of variants, particularly the predominance of P5‐II alongside P5‐I and P5‐III infections. In noncrop hosts, no association was observed between variant identity and symptom expression, which is likely to reflect the high frequency of asymptomatic infections in these species. Additional factors including cultivar, environmental conditions, and timing of infection further complicate relationships between variant identity and disease severity. As with other biological traits, virulence is likely to be influenced by multiple genomic regions rather than the P5 region alone. Coinfection may also alter transmission dynamics or facilitate infection of otherwise suboptimal hosts, as reported for other plant–virus systems.^65^ The high frequency of coinfection observed here highlights the need for further investigation into interactions among TuYV variants.

The P5 variant detected in *M. persicae* matched that of the host plant in ~50–77% of cases, with particularly strong concordance for P5‐II. Similar findings were reported by Pimenta *et al*.^66^ who observed a proportion of aphids carrying variants different from those detected in their host plants, likely reflecting recent movement between hosts. In the present study, adult aphids were selected to maximize RNA yield, but aphid morphs were not distinguished. Future studies should test alate and apterous individuals separately to better understand movement dynamics and transmission pathways. Additionally, variant concentrations may differ substantially between aphids and plants, potentially causing some infections to fall below the qPCR detection threshold at the time of sampling.

## 5 CONCLUSIONS

This study demonstrates that TuYV epidemiology in Australian *B. napus* crops is strongly influenced by noncrop reservoirs, seasonal aphid dynamics and agronomic practices. *Arctotheca calendula* and *R. raphanistrum* were identified as major reservoirs of both *M. persicae* and TuYV, with even low‐density populations likely to contribute to crop infection. Variant P5‐II predominated across hosts and regions, although coinfection among variants was common and may influence symptom development and transmission dynamics. Foliar disease symptoms were reliable indicators of infection in *B. napus* during spring but not in noncrop hosts, emphasizing the importance of molecular diagnostics in reservoir surveillance. Earlier sowing dates were associated with increased TuYV incidence, highlighting sowing time as a potentially important management tool.

Frequent TuYV epidemics over the past decade underscore the need to strengthen integrated disease management strategies in Australian production systems. As *M. persicae* populations continue to evolve resistance to insecticides,^67^, ^68^, ^69^, ^70^ greater reliance will likely be placed on newer chemistries, plant host resistance, and cultural and phytosanitary control measures.^71^, ^72^ Targeted weed management, strategic sowing, and enhanced surveillance of both aphids and virus reservoirs will be critical for reducing TuYV spread. Importantly, management strategies will need to account for regional differences between persistent ‘green‐bridge’ systems and the more seasonal ‘green‐ramp’ dynamics characteristic of Mediterranean environments.

## CONFLICT OF INTEREST

The authors declare no conflict of interest.

## Supporting information


**Figure S1.** Percentage cover of noncrop plant families across sampling seasons (autumn and spring), geographic regions (SE Australia and SW Australia) and years (2021 to 2023). For visualization purposes, only plant families comprising ≥25% of vegetation cover within a sampled plot are shown.
**Figure S2.** Predicted probability of *M. persicae* occurrence in noncrop plant families across sampling seasons (autumn and spring) and geographic regions (SE Australia and SW Australia). Significant pairwise comparisons are denoted as: *,*P* < 0.05; **, *P* < 0.01; ***, *P* < 0.001.
**Figure S3.** Predicted probability of TuYV occurrence in noncrop plant families across sampling seasons (autumn and spring) and geographic regions (SE Australia and SW Australia). Significant pairwise comparisons are denoted as: *, *P* < 0.05; **, *P* < 0.01; ***,*P* < 0.001.
**Figure S4.** Concordance of TuYV P5 variants detected in *M. persicae* and corresponding crop (*B. napus*) and noncrop plants. *n*‐values above each bar represent the total number of TuYV variants of each type detected in *M. persicae* across each plant type.
**Figure S5.** Observed (dark bars) and expected (light bars) counts of symptomatic and asymptomatic plants among TuYV P5 variants in crop (*B. napus*) and noncrop plants.

## Data Availability

All code and most data required to reproduce the analyses in this manuscript are available at Figshare repository 10.6084/m9.figshare.32654271. Coordinates of sampled sites are not shared to maintain privacy of growers who participated in the study.
