# Supplementary material for: Epidemiological drivers of turnip yellows virus and its vector Myzus persicae in Australian canola (Brassica napus) systems: a 3‐year study of commercial crops and noncrop vegetation
Source: Pest Manag Sci. 2026 Sep 20;82(11):10671–82. doi: 10.1002/ps.71325 (PMC13665651; doi:10.1002/ps.71325)
Supplement: Supplementary file 1 [file PS-82-10671-s001.docx]

**SUPPLEMENTARY MATERIAL**

**Epidemiological drivers of turnip yellows virus and its vector *Myzus persicae* in Australian canola (*Brassica napus)* systems: a 3-year study of commercial crops and noncrop vegetation**

Authors: Samantha E. Edley^1,2^, Owain R. Edwards^3,4^, Benjamin S. Congdon^5^, John G. Black^1,6^, Marielle Babineau^1,7^, Anthony R. van Rooyen^1^, Alex Slavenko^1^, Roger A. C. Jones^8^, Andrew R. Weeks^1^, and Paul A. Umina^1,9*^

^1^ Cesar Australia, 95 Albert Street, Brunswick, Victoria 3056, Australia

^2^ Agriculture Victoria, DEECA Mildura Office and Laboratory, Cnr Koorlong Avenue and Eleventh Street, Irymple, Victoria 3498, Australia

^3^ CSIRO Environment, Black Mountain, 147 Underwood Avenue, Floreat, Western Australia 6014, Australia

^4^ Entozyme Pty Ltd, 1/22 Stirling Highway, Nedlands, Western Australia 6009, Australia

^5^ Western Australian Department of Primary Industries and Regional Development, 14 Monash Gate, Jandakot, Western Australia 6164, Australia

^6^ In Vitro Technologies, 7-9 Summit Road, Noble Park North, Victoria 3174, Australia

^7^ Department of Microbiology and Immunology, School of Veterinary Sciences, The University of Melbourne, Parkville, Victoria 3010, Australia

^8^ UWA Institute of Agriculture, The University of Western Australia, 35 Stirling Highway, Perth, Western Australia 6009, Australia

^9^ School of BioSciences, Bio21 Institute, The University of Melbourne, Parkville, Victoria 3010, Australia

## Supplementary Methods

First-year field sample collections

Sampling was conducted during spring 2021 (early September to early October) at 30 sites in SW Australia (Western Australia; 15 targeted sites and 15 randomly selected) and 30 sites in SE Australia (Victoria and southern New South Wales [NSW]; 16 targeted sites and 14 randomly selected). At each site, one marker was positioned within non-crop vegetation adjacent to a west-facing *B. napus* crop edge, and a second marker was placed 100 m away along the crop edge, avoiding bare ground where possible. The crop border was used as a transect, with non-crop plants randomly sampled at 10 m intervals (*i.e.,* 10 plants between markers). An additional 1-5 non-crop plants known to be hosts of *M. persicae* and/or TuYV were also sampled along each transect, resulting in 11-15 plants per crop border. Floristic composition of the non-crop vegetation (recorded to species or genus level), the presence or absence of visual disease symptoms on plant leaves, and the percentage ground coverage of each non-crop plant species were recorded. Plant coverage was subsequently calculated for each plant family as a proportion of the total non-crop vegetation.

The foliage of each plant was thoroughly inspected for aphids, and abundance was categorised as 0, 1-10, 11-100, 101-1000, >1000 individuals per plant. Aphids were collected and separated by species into 1.5 ml screw top Eppendorf tubes containing 100% ethanol. Samples were stored at 4 °C during transport and transferred to -20 °C upon return to the laboratory.

Whole leaf samples were taken from each plant and placed in sealed zip lock plastic bags with paper towel and labelled. Samples were kept cool during transport and stored at 4 °C once back in the laboratory.

At all targeted sites (*n* = 31), the adjacent *B. napus* crops were also sampled in spring. Crop plants were sampled along a transect that was directly adjacent to, and mirroring, the non-crop vegetation transect, with the total number of crop samples corresponding to the number of non-crop samples collected. Similar to the non-crop vegetation assessments, we recorded the presence or absence of visual disease symptoms on plant leaves and categorised aphid abundance per plant. We also collected aphid and leaf samples as described above. Where present, younger and visibly stressed (wilted) leaves were preferentially sampled to maximise virus detection. Wherever possible, the sowing date and crop cultivar were recorded.

Second-year field sample collections

The second-year field sampling was similar to 2021, with some modifications.

In autumn 2022 (mid-April to late April), prior to crop emergence, sampling was conducted at 24 targeted sites in SE Australia within non-crop vegetation adjacent to fields scheduled to be sown with *B. napus*. These sites were located on farms with a known history of *M. persicae* and TuYV occurrence in canola crops from previous growing seasons.

In spring 2022 (early September to mid-October), sampling was conducted at 30 sites in SW Australia and 30 sites in SE Australia, once again spanning multiple rainfall zones. Similar to 2021, ~15 sites were targeted and ~15 randomly selected in each region, with the same criteria used for site selection, marker placement, and site characterisation. In SE Australia, the targeted spring sites were a sub-sample of the 24 sites sampled in autumn of that year.

Unlike 2021, sampling intensity within the non-crop vegetation was proportional to the percentage (basal) cover of non-crop hosts along each transect. Between 8 and 15 plants (~12 on average) were sampled per site, with a minimum of one plant sampled per host species present. Plant coverage was calculated for each plant family as a proportion of the total non-crop vegetation.

The foliage of each plant was thoroughly inspected for aphids. Rather than using the categorical abundance classes applied in 2021, aphid abundance was recorded as an exact count up to 20 individuals per plant, with counts exceeding 20 recorded as ‘20+’. This modification was made because most plants sampled in 2021 harboured fewer than 20 aphids, and the broader categorical approach failed to detect any relationship between aphid abundance and TuYV presence. Aphid collections, transport and storage once in the laboratory, followed the methods described above.

Leaf samples were taken from each plant, with younger, lower leaves preferentially selected. To do this, ~1 x 2 cm leaf sections were removed using metal forceps and scissors and placed individually into a 1.5 ml screw top Eppendorf tubes containing RNA*later*® (Thermo Fisher Scientific, USA). This method ensured samples could be stored for longer without RNA degradation. Between each sample, the forceps and scissors were submerged in 100% ethanol followed by flame sterilisation. Waxy or hairy leaves were crushed to increase RNA*later*® absorption. Samples were stored at room temperature during transport and in the laboratory.

At each site, *B. napus* crop plants were sampled in the same manner. As in 2021, crop plants were sampled along a transect that was directly adjacent to, and mirroring, the non-crop vegetation transect, with the total number and position of crop samples corresponding to the number and position of non-crop samples collected.

Third-year field sample collections

In 2023, sampling was conducted at 15 targeted sites in SW Australia and 15 targeted sites in SE Australia in autumn (late April to late May). Once again, this focused on the non-crop vegetation adjacent to fields that were to be sown with *B. napus*. In SW Australia, no *M. persicae* were detected during the autumn period, precluding analyses of autumn aphid occurrence data in that region. The same 15 sites within each region were sampled again in spring (mid-September to early October).

Methods closely followed those used in 2022, although sampling of the non-crop vegetation was restricted to the predominant host plants of *M. persicae* and TuYV identified during 2021 and 2022 surveys. These included *Brassica* species, *A. calendula*, *C. bursa-pastoris, M. parviflora, Medicago sativa, R. raphanistrum, Rumex crispus, S. arvensis, Sisymbrium officinale, Taraxacum officinale*, *Trifolium repens,* and *Trifolium subterraneum.* In SE Australia, the floristic composition and percentage ground cover of non-crop plant species were recorded, whereas these measurements were not undertaken in SW Australia due to time constraints.

Statistical analyses

Model specifications and best fitting models for the individual research questions are as follows:

1. To compare *M. persicae* and TuYV occurrence among plant families, the dataset was filtered to only include plant families with ≥ 20 observations (*n* = 2207; dataset 1), as families with fewer observations contained insufficient values for modelling fitting. Two GLMMs were fitted with response variables representing: (i) *M. persicae* occurrence, and (ii) TuYV occurrence. The best-supported models (*p* < 0.001) included plant family, season (autumn vs. spring) and region (SW Australia vs. SE Australia) as fixed effects, and their two-way interactions with season. The *M. persicae* model also included the two-way interaction between region and plant family.
2. To examine whether TuYV occurrence differed between crop and non-crop plants, analyses were restricted to spring samples (*n* = 2608; dataset 2), as autumn sampling occurred prior to crop emergence. Consequently, season was excluded as a covariate. The best-supported model (*p* < 0.001) included plant type (crop vs. non-crop) and region as fixed effects.
3. To determine whether autumn non-crop host presence influenced *M. persicae* and TuYV detection in spring *B. napus* crops, two GLMMs were fitted using dataset 2, with response variables representing: (i) *M. persicae* occurrence, and (ii) TuYV occurrence. Plant coverage was included as a predictor variable, with plant family and region as covariates.
4. To explore the occurrence of TuYV P5 variants, dataset 2 was filtered to only include TuYV+ samples for which variant identity was determined (*n* = 1578; dataset 3). A multivariate response model was fitted using variant as the response variable. The model included TuYV P5 variant as a fixed effect, all two-and three-way interactions between variant and other predictor variables (season, region, and plant type), and no intercept term. Three random effects were included to account for variance/covariance structure among: (i) individual plants, (ii) sites, and (ii) years, corresponding to the random effects included in other models. Model selection followed the procedure described above. The best-supported model (*p* < 0.001) included: (i) TuYV P5 variant, (ii) the two-way interaction between variant and region, and (iii) the two-way interaction between variant and plant type.
5. To assess whether TuYV variants detected in host plants were associated with those detected in *M. persicae* collected from the same plants, dataset 4 was further filtered to only include TuYV+ *M. persicae* and TuYV+ host plants with identified variants (*n* = 541; dataset 4). A χ^2^ contingency table test was run using the ‘chisq.test’ function with 10,000 Monte Carlo simulated replicates to compute *p*-values.
6. To assess whether TuYV detection in *M. persicae* was associated with TuYV detection in host plants, *M. persicae* data were linked to corresponding plant data from the same sites and sampled non-crop plants (*n* = 378; dataset 5). The main fixed effect was TuYV occurrence in the host plant, as a binary factor. Models included all other covariates, and non-crop plant family. The best-supported model (*p* < 0.001) included TuYV occurrence and season as fixed effects, and their two-way interaction.
7. To assess whether autumn *M. persicae* occurrence was associated with TuYV in spring *B. napus*, spring crop TuYV infection data was linked to autumn *M. persicae* occurrence data from corresponding sites (*n* = 150; dataset 6). Random effects did not improve model fit (*p* = 1.0), and therefore a single logistic model including autumn *M. persicae* occurrence as a predictor was fitted.
8. To assess whether visual disease symptoms were reliable indicators of TuYV infection, the full dataset was filtered to include only plants for which symptom data was recorded (*n* = 3266; dataset 7). The main fixed effect was symptom occurrence as a binary factor, with season and plant type included as covariates. Region was included as a random effect to account for potential observer bias arising from different personnel conducting fieldwork in SW Australia and SE Australia. The best-supported model (*p* < 0.001) included as fixed effects: (i) virus symptom (present or absent), (ii) season, (iii) plant type, and (iv) and the two-way interaction between symptom occurrence and plant type.
9. To assess whether the TuYV P5 variants were associated with differences in visual disease symptoms, dataset 7 was further filtered to include only TuYV+ host plants with identified variants (*n* = 1675; dataset 8). Chi-square contingency table tests were performed as described above.
10. To assess whether sowing date affected TuYV incidence of *B. napus* crops, the full dataset was filtered to include only those sites for which sowing date was known (*n* = 638; dataset 9). A GLMM was fitted with sowing date as the fixed effect and region as a covariate, and their two-way interaction. The best-supported model (*p* < 0.001) did not include the interaction term.

## Supplementary Figures

**Figure S1.** Percentage cover of noncrop plant families across sampling seasons (autumn and spring), geographic regions (SE Australia and SW Australia) and years (2021 to 2023). For visualisation purposes, only plant families comprising ≥25% of vegetation cover within a sampled plot are shown.

**Figure S2.** Predicted probability of *M. persicae* occurrence in noncrop plant families across sampling seasons (autumn and spring) and geographic regions (SE Australia and SW Australia). Significant pairwise comparisons are denoted as: *, *p* < 0.05; **, *p* < 0.01; ***, *p* < 0.001.

**Figure S3.** Predicted probability of TuYV occurrence in non-crop plant families across sampling seasons (autumn and spring) and geographic regions (SE Australia and SW Australia). Significant pairwise comparisons are denoted as: *, *p* < 0.05; **, *p* < 0.01; ***, *p* < 0.001.

**Figure S4.** Concordance of TuYV P5 variants detected in *M. persicae* and corresponding crop (*B. napus*) and noncrop plants. *n* values above each bar represent the total number of TuYV variants of each type detected in *M. persicae* across each plant type.

**Figure S5.** Observed (dark bars) and expected (light bars) counts of symptomatic and asymptomatic plants among TuYV P5 variants in crop (*B. napus*) and noncrop plants.
